# Quantitative Detection of Biological Nanoparticles Using Twilight Off-Axis Holographic Microscopy: Insights on Complex Formation between PEGylated Gold Nanoparticles and Lipid Vesicles

**DOI:** 10.1021/acs.jpcb.5c04228

**Published:** 2025-09-09

**Authors:** Julia Andersson, Anders Lundgren, Erik Olsén, Petteri Parkkila, Daniel Midtvedt, Björn Agnarsson, Fredrik Höök

**Affiliations:** 1 Department of Physics, Division of Nano and Biophysics, 11248 Chalmers University of Technology, Fysikgränd 3, Göteborg 41296, Sweden; 2 Department of Chemistry & Molecular Biology, University of Gothenburg, Medicinaregatan 7B, Göteborg 41390, Sweden; 3 Michael Smith Laboratories, University of British Columbia, 2185 East Mall, Vancouver, BC V6T 1Z4, Canada; 4 Department of Physics, University of Gothenburg, Origovägen 6b, Göteborg 41296, Sweden

## Abstract

The detection of biological nanoparticles (NPs), such as viruses and extracellular vesicles (EVs), plays a critical role in medical diagnostics. However, these particles are optically faint, making microscopic detection in complex solutions challenging. Recent advancements have demonstrated that distinguishing between metallic and dielectric signals with twilight off-axis holographic microscopy makes it possible to differentiate between metal and biological NPs and to quantify complexes formed from metal and biological NPs binding together. Here, this method is employed to investigate complex formation through specific interactions between streptavidin (StrAv)-modified gold NPs (StrAv-AuNPs) and large biotin-containing unilamellar lipid vesicles (biotin-LUVs), serving as virus and EV mimics. To minimize AuNP self-aggregation during functionalization of PEGylated 25 nm radius AuNPs with tetrameric StrAv, 0.06% biotin-PEG (∼5 biotin per AuNP) was used, which also serves to ensure that aggregation involving multiple LUVs is effectively prevented. While the StrAv-biotin ratio did not significantly affect AuNP self-aggregation upon fabrication of StrAv-AuNPs, a 1000-fold StrAv excess with respect to biotin-PEG on the AuNPs was required to fabricate StrAv-AuNPs with the anticipated reactivity with biotin-LUVs. Through a combination of waveguide scattering microscopy, surface plasmon resonance, and twilight off-axis holographic microscopy, we demonstrate that this likely stems from a dramatic reduction in the association rate constant between StrAv and biotin within the PEG layer. Furthermore, by using a mixture of 3 kDa nonbiotinylated PEG and 5 kDa biotin-PEG, functional StrAv-AuNPs were successfully fabricated at an orders of magnitude lower StrAv-to-biotin ratio, enabling a sub-pM limit of detection of biotin-LUVs using off-axis holography.

## Graphical abstract

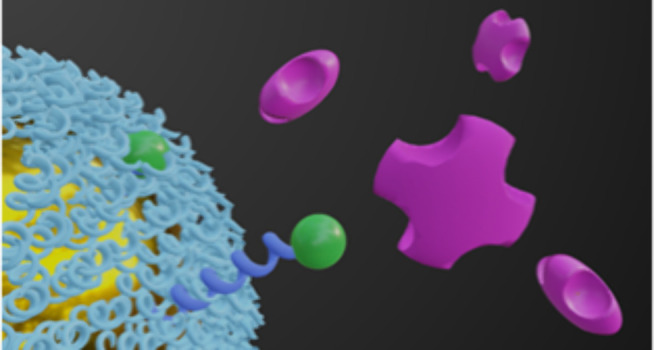

## Introduction

A central challenge both in diagnostic assays that rely on the application of gold nanoparticles (AuNPs) for readout and in therapies that use AuNPs for facilitating interventions is the precise control of the surface functionalization of the AuNPs. Functionalized AuNPs are widely employed for specific identification of cell surface markers,^1–3^ in cell- and tissue-specific drug delivery,^4–7^ as diagnostic contrast agents,^8,9^ and in photothermal theranostic applications.^10,11^ Due to their high optical contrast, AuNPs, when appropriately surface-functionalized with suitable recognition elements, offer high sensitivity in surface-based diagnostic systems^12,13^ and in assays based on color changes caused by plasmonic coupling between AuNPs brought in close proximity by biomolecular interaction.^14–16^ AuNPs containing multiple binding moieties can also facilitate complex formation between biological nanoparticles, such as viruses and extracellular vesicles.^17^ This interaction, however, results in an insignificant color change of the AuNPs but is instead detectable as a change in size, which can be measured, for example, using nanoparticle tracking analysis (NTA).^18^

It was recently demonstrated that off-axis holographic microscopy enables both to measure the hydrodynamic radius of complexes formed between AuNPs and faint biological NPs and to clearly differentiate between distinct types of complexes.^19^ This capability offers a promising approach for classifying suspended particle complexes composed of either pure metallic nanoparticles, of optically faint dielectric nanoparticles, or of hybrid mixtures thereof, at the single-particle complex level without relying on the spectral shift.^19^ This ability is particularly valuable for identifying sparse aggregates of hybrid particle complexes hidden in a background of manifold more nonreacted particles and sets off-axis holographic microscopy apart from NTA, which primarily provides information on particle size. Furthermore, since off-axis holography detection does not depend on spectral shifts originating from plasmonic coupling between adjacent AuNPs, it offers greater flexibility regarding the thickness of the surface functionalization layer on the AuNPs, whereas colorimetric assays are typically constrained by using very thin layers (on the order of a few nm) to facilitate detection through plasmonic coupling.^14–16^ Complex formation between different suspended NPs through biospecific interaction is, however, by itself a multifaceted process,^20,21^ strongly influenced by both the relative and absolute concentration of the different NPs as well as the concentration of the binding and recognition moieties tethered to their respective surfaces. Therefore, the optimal design of such assays requires exceptionally precise control over the surface functionalization of the NP probes.

Thiol-gold chemistry is an effective method for functionalizing AuNPs with biomolecular ligands. Low-molecular-weight ligands, such as peptides and nucleic acids, can, using this method, be straightforwardly and reliably tethered to AuNPs by taking advantage of intrinsic cysteines or thiol groups.^22,23^ However, complex biomolecules like proteins may often require more sophisticated attachment strategies to maintain their functional integrity and ensure optimal binding orientation.^24^ One effective and frequently applied approach is to first modify the AuNPs with an inert, thiol-modified polyethylene glycol (SH-PEG) layer.^25,26^ To accomplish protein coupling, the SH-PEG is typically mixed with SH-PEG that carries a reactive group for protein attachment.^27,28^ The inert PEG spacer, which can be made of different polymer chain lengths (molecular weights), enhances protein stability and accessibility, ensuring a more controlled protein presentation on the AuNPs. Often, each AuNP will carry multiple reactive PEG molecules that can each bind to multiple sites on the same proteins. This may, in turn, lead to AuNP self-aggregation during fabrication. Therefore, both the surface density of functional groups on the AuNPs and the relative concentration of AuNPs and proteins, as well as mixing conditions, must be carefully controlled to achieve AuNP–protein conjugates with a proper functionality without inducing AuNP aggregate formation.

To investigate the formation of complexes between streptavidin (StrAv)-modified AuNPs and biotin-modified lipid vesicles using off-axis holography, particular emphasis was placed on the functionalization of biotin-PEG-modified AuNPs (biotin-AuNPs) with StrAv to avoid AuNP self-aggregation during the conjugation reaction, a challenge arising from the tetravalent biotin-binding capacity of StrAv.^29^ Precise control over this functionalization step is critical to prevent undesired AuNP aggregation but also to ensure the intended reactivity of the AuNP probes, which was verified through waveguide scattering microscopy (WGSM) and, for planar gold, using surface plasmon resonance (SPR) measurements. StrAv-induced aggregation of biotin-AuNPs was assessed by NTA and off-axis holography measurements for different ratios of biotin-PEG moieties on the suspended AuNPs to StrAv in solution. In particular, by utilizing the discriminative power of off-axis holographic microscopy to resolve different nanoparticle complexes, we evaluated the capacity of StrAv-modified AuNPs (StrAv-AuNPs) to detect dielectric biotin-LUVs in the presence of a substantial excess of AuNPs, which was deliberately employed to facilitate rapid reaction kinetics.

The study highlights the benefit of using biotin-PEG with a higher molecular weight compared to the surrounding nonfunctionalized PEG for controlled functionalization of AuNPs with a low StrAv coverage and the importance of sustaining controlled conditions for StrAv and biotin-AuNP mixing during the reaction. The material-specific fingerprints of complexes formed from AuNPs and optically faint LUVs provided by off-axis holography show that precisely functionalized StrAv-AuNPs can offer sub-pM detection of biotin-modified LUVs, serving as a model for viruses and extracellular vesicles. Specifically, due to the unique signal obtained for complexes formed between metal nanoparticles and lipid vesicles, the assay is compatible with the use of a high concentration of AuNPs, which accelerates the complex formation reaction without formation of aggregates containing multiple LUVs leading to completion within tens of minutes. Under similar conditions, detection of complex formation is shown to be obscured using conventional size determination methods like NTA and UV–Vis-based colorimetry.

## Results and Discussion

The surface of ∼25 nm radius AuNPs was first functionalized by incubating AuNPs with 10/nm^2^ SH-PEG­(5k) and SH-PEG­(5k)-biotin at a ratio of 1575:1, to provide on average around 5 biotin-PEG per AuNP.^30^ This modification increased the hydrodynamic radius of the AuNPs by approximately 20 nm relative to the unmodified AuNPs (Figure 1a and Figure S3).^30^ Following the removal of unreacted SH-PEG, the biotin-PEG­(5k)-AuNPs were gradually added to a stirred suspension of StrAv (1.7 μM) in phosphate buffered saline (PBS, pH 7.4) using a positive displacement pump until a final StrAv-to-biotin ratio of 1000:1 was achieved. A large excess of StrAv was used to suppress aggregation of biotin-PEG­(5k)-AuNP due to the tetravalent biotin-binding capacity of StrAv.^20,21,29^ The low degree of aggregation was confirmed by comparing the size distributions of biotin-PEG­(5k)-AuNPs and StrAv-biotin-PEG­(5k)-AuNPs after the removal of excess StrAv (Figure 1b), which revealed similar polydispersity indices. The observed ∼7 nm reduction in the mean hydrodynamic radius of StrAv-AuNPs is attributed to the salt-induced contraction of PEG in PBS compared to pure water, as previously reported.^31^

**Figure 1:**
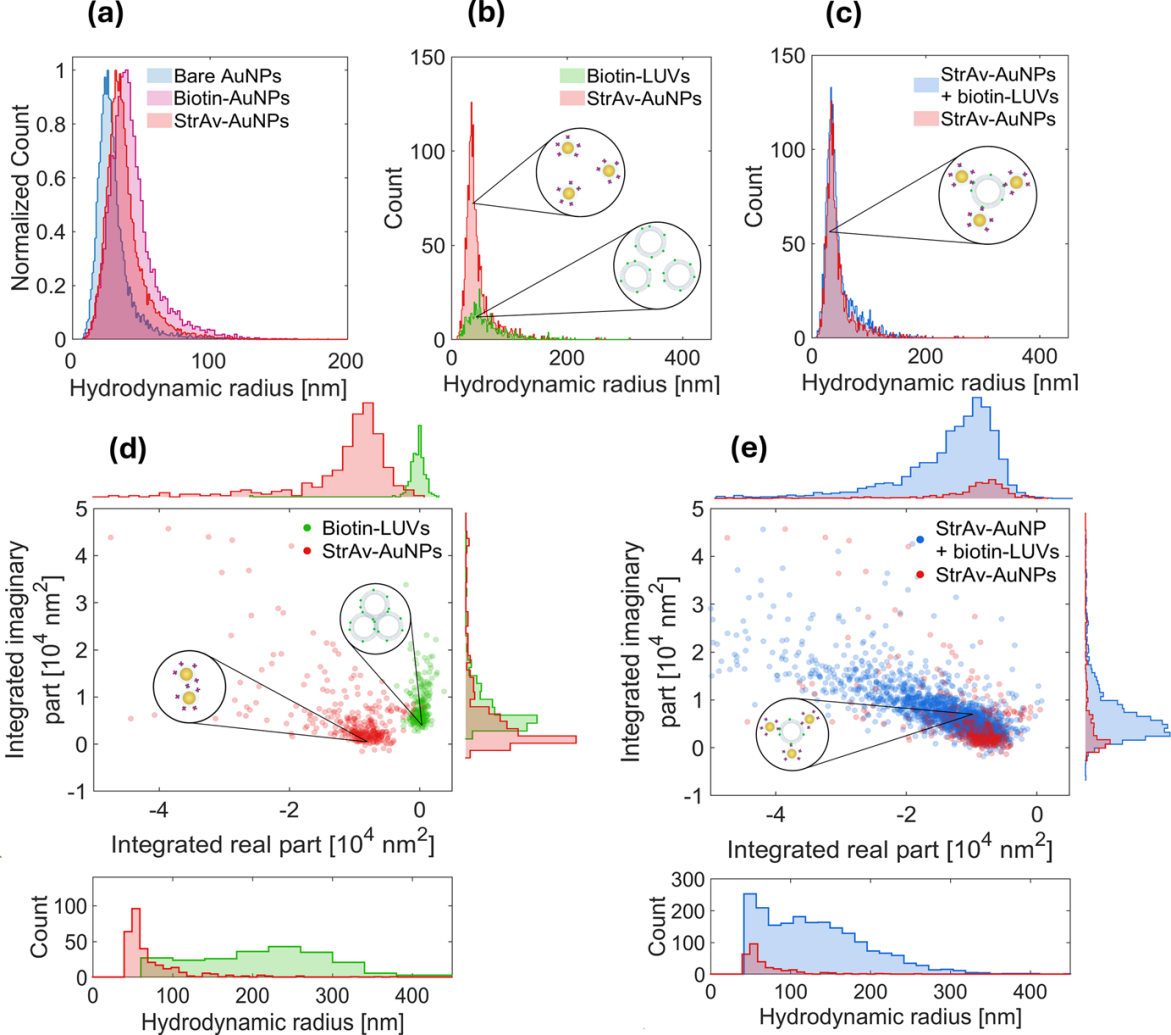
Detection of complex formation between StrAv-AuNP and biotin-modified vesicles using nanoparticle tracking analysis (NTA) and twilight off-axis holographic microscopy. (a) NTA-based determination of the hydrodynamic radius distribution of bare AuNPs (blue), AuNPs modified with 5k PEG and 5k PEG-biotin (biotin-PEG­(5k)-AuNPs) (pink) at a molar ratio of 1564:1 (corresponding to ∼5 biotin per AuNP), and StrAv-AuNPs (red) modified with StrAv measured after slow addition of biotin-AuNPs to a StrAv suspension at a final AuNP-to-StrAv ratio of 1000:1. (b, c) NTA-based determination of the hydrodynamic radius distribution of StrAv-AuNPs (red) and biotin-LUVs (green) measured (b) separately at concentrations of approximately 4 × 10^8^/mL and 3 × 10^6^/mL, respectively, and (c) after mixing and 30 min of incubation at a StrAv-AuNP:biotin-LUV ratio of 132:1. (d, e) Scatter representations of the integrated imaginary part of the off-axis holographic microscopy signal plotted versus the corresponding integrated real part for each individual detection, with the data in (d) and (e) representing the samples analyzed in (b) and (c), respectively. Also shown in (d) and (e) are the corresponding hydrodynamic radius distributions determined for the detections made using off-axis holographic microscopy. The insets represent schematic illustrations of the anticipated detection in each suspension. To aid a comparison, the data for StrAv-AuNPs are shown in red in all panels.

To investigate complex formation between StrAv-AuNPs and biotin-LUVs, StrAv-AuNPs (6.6 × 10^10^/mL) were subsequently mixed with ∼75 nm radius biotin-LUVs at a two orders of magnitude lower concentration (5 × 10^8^/mL). The LUVs contained 1 mol% biotinylated lipids (Figure 1b), corresponding to approximately 1000 surface-exposed biotin moieties per vesicle. The mixture was incubated for 30 min at room temperature to allow for specific binding between the StrAv-AuNPs and biotin-LUVs to take place, thereby inducing the formation of AuNP–LUV complexes, and potentially the formation of higher-order aggregates through bridging interactions,^18^ although this process is expected to be minimal due to the intentional limitation of StrAv probe molecules on the AuNPs. After the incubation, the suspension was analyzed by using both NTA and off-axis holographic microscopy. To comply with the recommended operating range of the NTA Instrument, the sample was diluted 167-fold prior to measurement.^18^ NTA revealed a size distribution and modal diameter nearly identical with those of unbound AuNPs (Figure 1c), suggesting that the suspension remained dominated by free, monomeric StrAv-AuNPs, even after complex formation (Figure 1b). This can be attributed to the two orders of magnitude higher StrAv-AuNP concentration relative to biotin-LUVs, resulting in an insignificant reduction in the concentration of monomeric StrAv-AuNPs, insufficient to influence the measured size distribution. This observation is consistent with previous use of NTA to follow metallic nanoparticle-induced interlinking of lipid vesicles, which requires careful fine-tuning of the ratio between the AuNP probes and target NPs, which, in turn, slows down the reaction rate at low concentrations and complicates detection of optically faint target particles in unknown samples. Similarly, the high StrAv-AuNP:biotin-LUV ratio, combined with the large interparticle distance of StrAv-AuNPs in complex with biotin-LUVs, prevents detection of complex formation by plasmonic coupling (Figure S4).

We recently demonstrated that off-axis holographic microscopy is capable of distinguishing metal NPs and dielectric NPs through the complex-valued optical signal.^19^ This approach provides unique optical signatures for metal NPs, dielectric NPs, and hybrid clusters formed between metal and dielectric NPs and may thus help circumvent the limitation of both NTA and UV–Vis spectroscopy, particularly when only a minor fraction of metal NP probes participate in the reaction generating detectable contrasts, as is typically the case under conditions of a high excess of metal NPs.

In brief, the integrated off-axis holography signal from a detected particle, *E*_p_, is related to its complex-valued polarizability as

$$\int E_{p}/|E_{0}|dx=ik\alpha /2$$ 1

where *k* is the wavenumber of the illuminating light and *E*_0_ is the complex-valued optical field of unscattered light. The polarizability is given by

$$\alpha =3V\frac{n_{p}^{2}-n_{m}^{2}}{n_{p}^{2}+2n_{m}^{2}}$$ 2

where *n*_p_ and *n*_m_ are the refractive indices of the particle and medium, respectively, and *V* is the particle volume. In this way, the real and imaginary parts of the holography signal are proportional to the imaginary and real parts of the particle refractive index, relating to particle absorption and particle scattering, respectively. Specifically, the real part of the off-axis holography signal from a detected particle *E*_p_ is related to the absorption cross section σ as

∬Re(EpE0)dA=−σ2 3

while the integrated imaginary part of the signal is given by

∬Im(EpE0)dA≈2πλ0VΔn 4

where λ_0_ is the vacuum wavelength of light and Δ*n* = *n*_p_ – *n*_m_ is the refractive index difference between the particle refractive index *n*_p_ and the refractive index of the medium *n*_m_.^19^ Since the refractive index of dielectric particles has a negligible imaginary part, it follows that the holography signal emanating from a dielectric particle will have a negligible real part. AuNPs, on the other hand, have a refractive index with a considerable imaginary part at the illumination wavelength used in this work (532 nm), and their holography signal will therefore also have a real part contribution.^19,32,33^

This is illustrated in Figure 1d, showing off-axis holographic microscopy data for individual suspensions of StrAv-AuNPs (6.6 × 10^10^/mL) and biotin-LUVs (5 × 10^8^/mL) measured separately. The detections obtained for StrAv-AuNPs emerge around integrated imaginary/integrated real off-axis holography signals of 0.67 × 10^4^/–1.19 × 10^4^ nm^2^ and have a modal radius around 50 nm. Corresponding detections obtained for biotin-LUVs cluster around 0.87 × 10^4^/–0.0003 × 10^4^ nm^2^ and have a modal radius of around 220 nm. The measured radii are thus two and three times the modal radii obtained from NTA measurements for StrAv-AuNPs and biotin-LUVs, respectively (Figure 1b). This suggests that objects detected with off-axis holography are aggregates rather than individual AuNPs and individual LUVs. Indeed, based on the integrated real part of the holographic microscopy signal and the absorption cross section of AuNPs, most detected StrAv-AuNP aggregates are likely dimers.^19^

No aggregate formation was observed in off-axis holographic microscopy measurements made on bare biotin-PEG­(5k)-AuNPs prior to StrAv functionalization (Figure S1). It is, therefore, reasonable to assume that the StrAv-AuNP aggregates form when mixing tetravalent StrAv and biotin-PEG­(5k)-AuNPs. Notably, the number of detected objects observed in the StrAv-AuNP sample corresponds to a concentration of 2.4 × 10^6^/mL, which is roughly 0.01% of the StrAv-AuNP concentration as determined using NTA (Figures 1a–c). This difference is attributed to the lower sensitivity of off-axis holography in detecting small nanoparticles compared with directed scattering detected with NTA.

When similar off-axis holographic microscopy analysis was performed for the sample containing a mixture of StrAv-AuNPs and biotin-LUVs, a 20-fold increase in the number of detected objects was observed compared to that when the StrAv-AuNPs were analyzed separately (Figure 1e). In the histograms of Figure 1e, the positions of these emerging objects reflect a significant contribution of both the integrated real part and the integrated imaginary part of the optical signal. Thus, the detected objects are presumably complexes containing both AuNPs and LUVs. While off-axis holography is insensitive to single StrAv-AuNPs and biotin-LUVs, having too low optical contrast to be detected, the cluster formation between StrAv-AuNPs and biotin-LUVs generates a unique signal above the detection threshold, which is not limited by the relatively high AuNP concentration, as was the case in the NTA measurements (cf. Figure 1c).

Although it is clear from Figure 1e that the complex formation between StrAv-AuNPs and biotin-LUVs generates a different signal than that of bare StrAv-AuNPs, the detections originating from pure StrAv-AuNPs and from StrAv-AuNPs bound to LUVs still overlap partly (Figure 1e), which may disturb the possibility of detecting low concentrations of biotin-LUVs. A previous study on StrAv-induced aggregation of biotin-modified AuNPs showed that the process is highly sensitive to the relative concentration of StrAv and biotin-modified AuNPs.^29^ To avoid StrAv-induced interlinking, we therefore prepared the StrAv-AuNPs by gradually adding biotin-PEG­(5k)-AuNPs to a StrAv solution containing a 1000-fold excess of StrAv relative to the final concentration of biotin. Assuming that there are approximately five biotins available per AuNP,^30^ this means that the StrAv-to-biotin-PEG­(5k)-AuNP ratio was always higher than 5 × 10^3^. This is two orders of magnitude higher than the ratio previously found effective in reducing aggregate formation,^34^ and accordingly, a too low concentration of StrAv is not a likely explanation for the aggregation formation observed upon StrAv functionalization of biotin-PEG­(5k)-AuNPs.

We instead hypothesized that the aggregate formation might result from the high StrAv concentration (1.7 μM) used, as this could increase the number of trace-level contaminants in the protein sample, potentially causing these very minor (∼0.01% of the StrAv-AuNP concentration) but undesired detections in these high sensitivity measurements. To investigate whether a reduced concentration of StrAv during biotin-PEG­(5k)-AuNP modification leads to reduced aggregation, the mixture containing a 1000-fold excess of StrAv was compared to mixtures prepared using either a 100-fold or a 10-fold excess of StrAv. NTA measurements cannot distinguish between these samples (Figure 2a); yet, it is clear from the off-axis holography readings that the previously observed aggregate formation remains and does not vary significantly between the samples (Figure 2b–d, red data points). The estimated concentrations of the detected StrAv-AuNP aggregates are 3.8 × 10^6^/mL, 4.1 × 10^6^/mL, and 2.4 × 10^6^/mL, at 10-fold, 100-fold, and 1000-fold StrAv excess, respectively, corresponding to roughly 0.01% of the total concentration of AuNPs in the sample.

**Figure 2:**
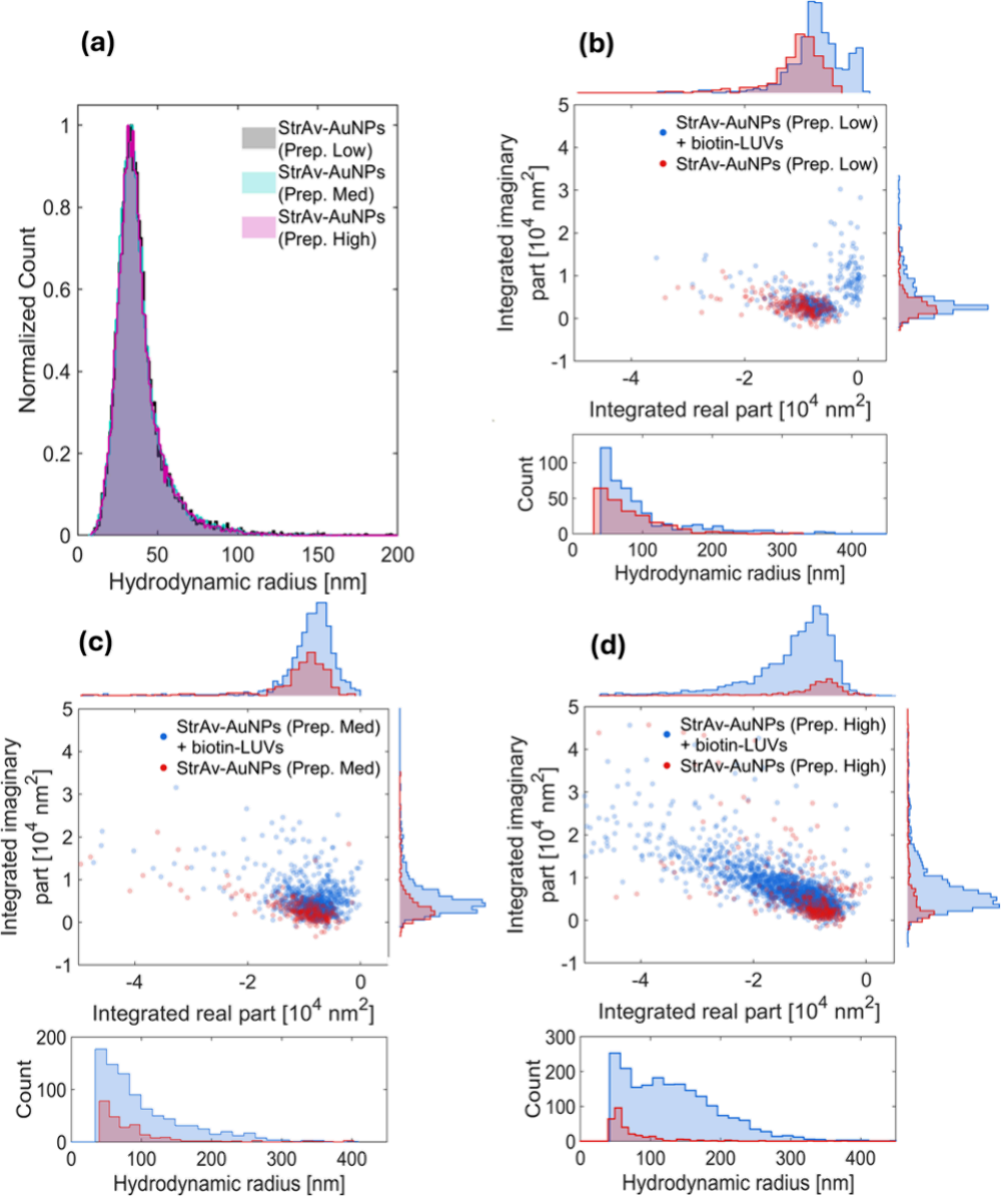
Analysis of StrAv-modified AuNPs at varying StrAv:biotin ratios. (a) NTA-based determination of the hydrodynamic radius distribution of StrAv-AuNPs functionalized through slow addition of StrAv to a AuNP suspension at final AuNP-to-StrAv ratios of 10:1 (gray, Prep. Low), 100:1 (cyan, Prep. Med.), and 1000:1 (pink, Prep. High). (b–d) Scatter representations of the integrated imaginary part of the off-axis holographic microscopy signal plotted versus the corresponding integrated real part for each individual detection. Also shown in (b–d) are the corresponding hydrodynamic radius distributions determined for the detections made using off-axis holographic microscopy.

At first sight, this result suggests that biotin-PEG­(5k)-AuNPs can be modified with StrAv also at lower StrAv-to-biotin-PEG­(5k)-AuNPs ratios without inducing pronounced aggregation through the StrAv-induced interlinking of biotin-modified AuNPs. It was, however, surprisingly observed that when StrAv-AuNPs were modified at ratios below 1000:1 StrAv per AuNP-bound biotin, their capability to induce complex formation with biotin-LUVs was significantly reduced; upon mixing with biotin-LUVs, StrAv-AuNPs made with 1000-fold excess of StrAV gave a 20-fold increase in the number of detected objects (Figure 2d, blue data points, Prep. High), while those made with 100-fold excess gave a 4-fold increase (Figure 2c, Prep. Med.) and those made with 10-fold excess gave only a 2-fold increase (Figure 2b, Prep. Low), compared to the signals obtained for StrAv-AuNPs only (Figure 2b–d, red data points). This suggests that a high excess of StrAv is required for successful modification of biotin-PEG­(5k)-AuNPs, despite the fact that even the lowest applied StrAv concentrations (>10 nM) can be considered high given the kinetic specifics of the interaction between StrAv and biotin.^35^

To evaluate the cause of the surprisingly low reactivity of StrAv-AuNPs modified at StrAv-to-biotin ratios of 100 and 10, respectively, the binding of the three differently modified StrAv-AuNPs to a flat biotin-functionalized surface was further inspected with surface-sensitive waveguide scattering microscopy (WGSM), which offers the possibility to measure real-time kinetics of AuNPs binding to surfaces with single AuNP resolution.^36^ The silica sensor surface of a WGSM chip was sealed with a microfluidic channel and modified with a 90:10 ratio of PLL-*g*-PEG:PLL-*g*-PEG-biotin^37^ to create a surface that displays a high concentration of biotin toward a nonadhesive background of PEG. A 10 μL liquid plug of StrAv-AuNPs (1.5 × 10^9^/mL) was injected at a flow rate of 10 μL/min, and time-resolved visualization of StrAv-AuNP binding events was imaged for around 10 min. The binding rates (AuNPs/s) were analyzed by counting individual binding events taking place within a field of view of 220 × 220 μm^2^ in the center of the flow channel. The binding rates range from 4 AuNPs/s for the StrAv-AuNPs prepared with a high (1000:1) excess to 0.4 and 0.2 AuNPs/s for those prepared with medium (100:1) and low (10:1) StrAv excesses, respectively (Figure 3a). The significantly lower rates of binding observed for StrAv-AuNPs fabricated using lower StrAv excesses indicate considerably lower StrAv coverage on these nanoparticles, a result that is consistent with the low level of complex formation observed using off-axis holographic microscopy data (Figure 2), suggesting that a high StrAv excess is indeed necessary to completely functionalize biotin-PEG­(5k)-modified AuNPs.

**Figure 3:**
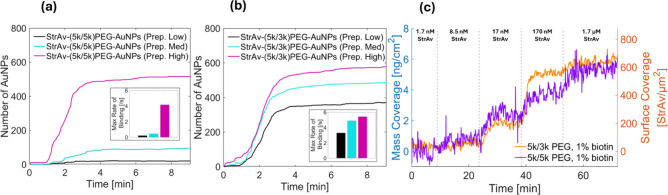
Capturing efficiency of StrAv-modified AuNPs and StrAv to bioitin-modified planar substrates analyzed using waveguide scattering microscopy (WGSM) and surface plasmon resonance (SPR). (a) Number of individual scattering detection events versus time upon injection of StrAv-(5k/5k)­PEG-AuNPs to a biotin-PLL-*g*-PEG-modified WGSM sensor surface for StrAv-bioitin-(5k)­PEG:(5k)­PEG-AuNPs functionalized with StrAv using StrAv:biotin ratios of 10:1 (black, Prep. Low), 100:1 (blue, Prep. Med.), and 1000:1 (magenta, Prep. High) (cf. Figure[Fig fig2]). (b) Same type of data as in (a) for StrAv-(5k/3k)­PEG-AuNPs. Insets in (a) and (b) display the rate of AuNP binding. Prior to functionalization with StrAv, the AuNPs were modified with 0.06% biotin-(5k)­PEG-SH mixed with either (a) (5k)­PEG-SH or (b) (3k)­PEG-SH, corresponding to ∼5 biotin moieties per AuNP. (c) Change in StrAv surface coverage versus time upon consecutive additions of StrAv at 1.7, 8.5, 17, 170, and 1700 nM to an SH-PEG-biotin-modified gold SPR sensor surface modified with biotin-(5k)­PEG mixed at a 1:99 ratio with (5k)­PEG (purple) and (3k)­PEG (orange). The StrAv surface coverage was estimated as described in references.^56,57^

Direct comparisons between binding reactions taking place onto planar and highly curved nanoparticle surfaces should be made with caution, yet it has been shown that StrAv binding to biotin-lipid containing supported lipid bilayers can reach saturation within tens of minutes when reacted with StrAv at concentrations down to 20 nM,^38^ which is comparable to the lowest concentration used in this study for modification of the AuNPs. Further, numerous studies have reported efficient StrAv binding when applying concentrations ranging between 100 and 200 nM to planar gold surfaces modified with various combinations of inert SH-PEG and different amounts of SH-PEG-biotin,^39,40^ which is comparable to the medium concentration used here.

Yet, many of these investigations and early studies on thiol-based surface modifications of AuNPs were primarily motivated by a desire to optimize colorimetric assays utilizing aggregation-induced near-field plasmonic coupling.^41^ These efforts therefore typically focused on functionalization protocols using tethering strategies based on significantly lower-molecular-weight molecules (0.3–1 kDa) than the 5 kDa PEG employed in our study.^42^ In contrast, the superiority of high-molecular-weight PEG in preventing nonspecific biomolecular binding^43^ has led to its predominant use and optimization in various therapeutic applications of AuNPs.^44,45^ In both cases, the primary strategy has been to optimize the density of functional groups on the AuNP surface, either to promote efficient aggregation in colorimetric assays or to enhance specific cellular targeting and uptake in therapeutic applications.^44^ For these modification strategies, it is well-established that extending the reactive groups beyond the inert background facilitates their accessibility and reactivity.^46–49^ In the case of high-molecular-weight PEGs, applications have been focused on optimizing the ratio between inert and functional groups to optimize the avidity of targeted nanoparticles requiring multiple weak interactions for strong binding, in most cases measured indirectly from their uptake into cells.^48–52^ Comparatively less focus has been put on precisely controlling the functionalization of AuNPs by employing a low fraction of functional PEG.^30^ To assess whether the inert high-molecular-weight PEG used in this work could indeed introduce significant steric hindrance also for the high-affinity interaction between StrAv and biotin, thus potentially obstructing its access to and binding with biotin-PEG on AuNPs at the low (0.06%) biotin-PEG coverage used in our modifications, we prepared AuNPs functionalized with a mixture of 3 kDa SH-PEG and 5 kDa SH-PEG-biotin (5k/3k) at a 1575:1 molar ratio, yielding approximately 5 biotin moieties per AuNP. We then performed StrAv modification at the same StrAv-to-biotin ratios as previously described, enabling a direct evaluation of whether steric hindrance from the high-molecular-weight PEG background limits streptavidin’s access to biotin sites under these conditions.^42^

A WGSM analysis of StrAv-AuNPs prepared in this way revealed a binding rate of 5.4 events per second for StrAv-(5k/3k)­PEG-AuNPs when functionalized with a high (1000:1) streptavidin excess (Figure 3b), comparable to that observed for StrAv-(5k/5k)­PEG-AuNPs (Figure 3a). Notably, while the highest rate of streptavidin-AuNP binding was observed for high streptavidin excess, the binding rates for StrAv-(5k/3k)­PEG-AuNPs modified at medium (100:1) and low (10:1) excesses were significantly higher (4.9 and 3.3 AuNPs/s) compared to the binding rates measured for StrAv-(5k/5k)­PEG-AuNPs modified under the corresponding conditions.

We also conducted an analysis to discern whether the observed rate of binding is primarily controlled by global diffusion (mass transport) or the reaction rate between StrAv and biotin on the sensor surface. For nanoparticles with diffusion constant *D* and concentration *C*, the time dependence of the diffusion-limited initial rate of binding to the floor of a rectangular flow cell can be expressed as^53^

$$\Delta \Gamma \left(t\right)=\xi {\left(D^{2}Q\right)}^{1/3}C\Delta t$$ 5

where *Q* is the volumetric flow rate and ξ is a constant related to the geometry of the flow cell:^54^

$$\xi =0.98{\left(\frac{2}{h^{2}wl}\right)}^{1/3}$$ 6

where *h*, *w*, and *l* are the height (35 μm), width (2 mm), and length (8 mm) of the channel, respectively. Given these expressions and the conditions of our experiment (see Materials and Methods), the anticipated diffusion-limited binding rate is 65 AuNPs/s. This rate is around one order of magnitude higher than the highest binding rate observed for StrAv-(5k/3k)­PEG-AuNPs (5.4 AuNPs/s), suggesting that the binding rate is indeed reaction-controlled despite the high affinity between streptavidin and biotin.

Assuming that for AuNPs fabricated with high StrAv excess, all five biotin moieties are occupied by StrAv, our measurements indicate that the reduced reactivity of StrAv-AuNPs produced at lower StrAv excess is due to a decreased average number of StrAv per AuNP. As was previously observed under similar experimental conditions, the rate of binding scales linearly with the average number of StrAv per AuNP.^30^

A reduction of the binding rate by more than a factor of 5 (the maximum number of StrAv per AuNP), which was observed when comparing StrAv-(5k/5k)­PEG-AuNPs produced at medium and low excess StrAv to those made with high StrAv excess, thus suggests that many of those AuNPs modified at low and medium excess of StrAv have no StrAv attached. This interpretation is also consistent with the observation that many of those NPs traversed the field of view without binding in the WGSM measurement, an observation that was only infrequently observed with the other StrAv-AuNPs (Supporting Information, Videos S1–S6). A strongly concentration-dependent binding of StrAv to planar gold surfaces modified with the same type of (5k/5k)­PEG-thiol and (5k/3k)­PEG-thiol modifications was also verified using SPR (Figure 3c). However, we refrain from attempts to directly compare the protein coverage on the planar gold to that on the highly curved AuNPs because of the significant differences in surface morphology.

As seen in Figure 4a–c (blue data points), off-axis holography data also show distinctive signals in both the integrated real and integrated imaginary parts for all the three variants of StrAv-(5k/3k)­PEG-AuNPs (6.6 × 10^10^/mL, 110 pM) when these were reacted with biotin-LUVs (5 × 10^8^/mL, 830 fM), which is consistent with successful StrAv functionalization for all tested ratios of StrAv to biotin-(5k/3k)­PEG-AuNPs according to WGSM (Figure 3b). In fact, the number of detections increases by factors of 12.6, 10.8, and 25.7 for StrAv-(5k/3k)­PEG-AuNPs modified at high, medium, and low StrAv excesses compared to the number of detection originating from self-aggregated StrAv-(5k/3k)­PEG-AuNPs, respectively, which also display a slightly reduced tendency to self-aggregate compared with their 5k/5k counterparts. Notably, this pronounced contrast was achieved even at sub-pM concentrations of lipid vesicles, underscoring the sensitivity and specificity of the approach. Furthermore, while a high StrAv excess is required during the fabrication of StrAv-(5k/5k)­PEG-AuNPs to detect the presence of biotin-LUVs, the use of StrAv-(5k/3k)­PEG-AuNPs not only results in an increased contrast in the detection signal but also reduces the need for excessive protein consumption during the functionalization process.

**Figure 4:**
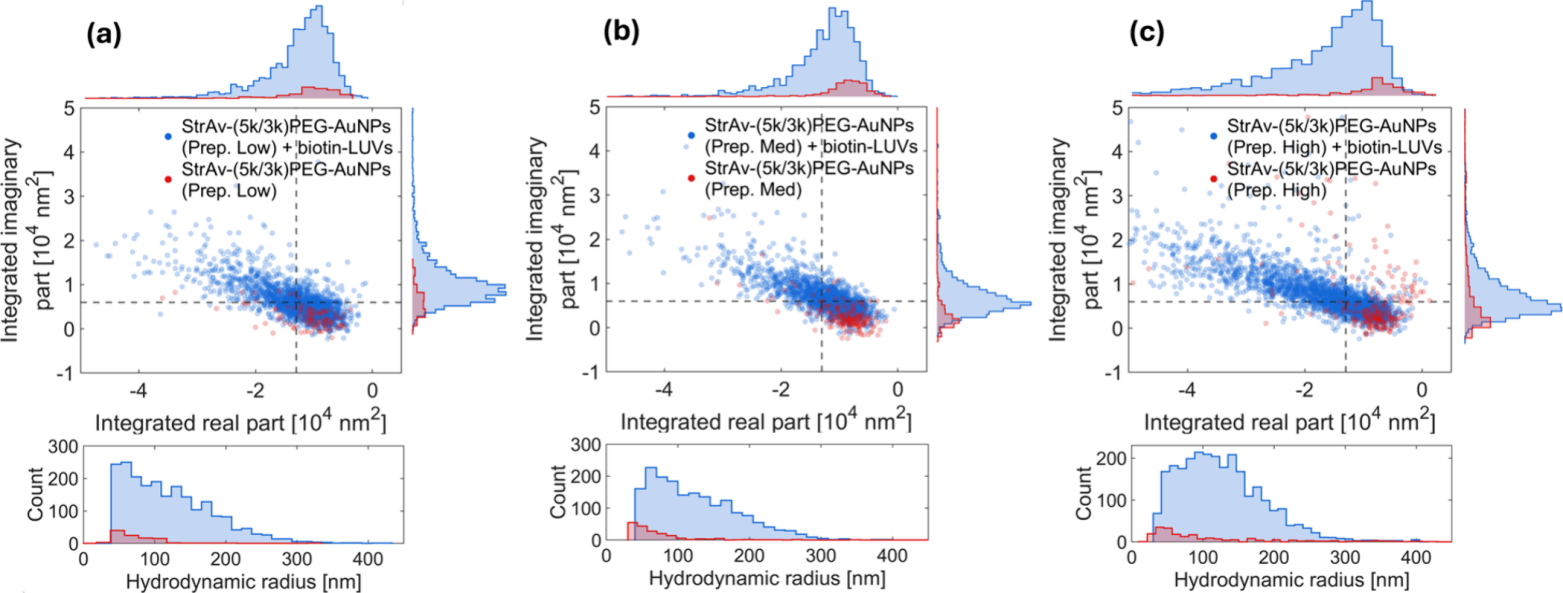
Twilight off-axis holographic microscopy evaluation of 100 nm biotin-LUV detection using StrAv-modified AuNPs PEGylated with 3 kDa thiol-PEG and 0.06% 5 kDa thiol-PEG-biotin (StrAv-(5k/3k)­PEG-AuNPs). (a–c) StrAv-(5k/3k)­PEG-AuNPs functionalized with StrAv at StrAv:biotin ratios of (a) 10:1, (Prep. Low), (b) 100:1, (Prep. Med.), and (c) 1000:1, (Prep. High) levels were observed in the absence (red) and presence (blue) of biotin-modified LUVs. Dashes indicate gating thresholds to identify the detections.

## Conclusions

This study demonstrates the efficiency of off-axis holography to offer rapid detection of specific complex formation between StrAv-modified AuNPs and biotin-LUVs in the presence of high excess monomeric StrAv-AuNPs at sub-pM biotin-LUV concentrations (Figure 4), in which case neither NTA (Figure 2a) nor UV–Vis spectroscopy (Figure S4) provides detectable contrasts. The results indicate that for optimal functionalization of AuNPs with StrAv, the biotin ligand should be attached to a PEG with a notably higher molecular weight than the unmodified PEG serving as an inert background. Specifically, biotin-PEG­(5k) combined with PEG­(3k) provided efficient StrAv binding at concentrations down to 17 nM (corresponding to a StrAv excess over biotin of 10) when grafted onto 25 nm radius AuNPs under conditions corresponding to 5 biotin-PEG per AuNP, while PEG­(5k) required a 1000-fold excess of StrAv. This observation should be considered in the context of the polydispersity of the SH-PEG and SH-PEG-biotin used, which had polydispersity indices (PDI) of approximately 1.02. Assuming normal distribution dispersity, in which case $\mathrm{PDI}=1+{\left(\frac{\sigma }{\mu }\right)}^{2}$ , where μ and σ are the mean molecular weight and variance, respectively, the variance corresponds to 0.7 kDa for PEG with a mean molecular weight of 5 kDa. Considering that the height of end-grafted PEG onto a surface is around 10 nm and that the extension of individual PEG molecules roughly scales with molecular weight, the variability in PEG extension may exceed 2 nm. Potentially, this is enough to hinder the accessibility of the 0.25 kDa biotin moiety in a way that prevents its attachment into the StrAv binding pockets, despite their high affinity. The association rate constant for the interaction between biotin and StrAv has been reported to be as high as 10^7^ M^–1^ s^–1^.^35^ We observed a strong transition in binding of StrAv-to-biotin-PEG­(5k/5k)-AuNPs between 0.17 and 1.7 μM StrAv. The absence of StrAv binding at 0.17 μM after more than 1 h of incubation thus suggests a reduction in the association rate constant by several orders of magnitude, which is likely attributed to steric hindrance and slow dynamics of PEG in the stretched brush configuration adopted on gold.^43^ This observation merits consideration in the context of prior studies employing inert and functionalized high-molecular-weight PEGs to optimize the multivalent attachment to cellular surfaces. In these cases, it is often challenging to discern whether the rate-limiting step for the reaction is the formation of an initial bond or the establishment of subsequent bonds. The significant reduction by several orders of magnitude in the association rate constant for the high-affinity interaction between streptavidin and biotin indicates that for PEGylated gold nanoparticles engineered for multivalent interactions, the formation of the first bond constitutes the primary kinetic barrier.

Considering the off-axis holographic detection scheme utilized in this work, the contribution from clusters formed between StrAv-AuNPs and biotin-LUVs to both the integrated real and integrated imaginary parts of the signal provides a unique fingerprint, distinguishing it from methods that rely on changes in size, such as dynamic light scattering (DLS) or NTA, or spectral changes, typically probed using UV–Vis spectroscopy. However, a small fraction of the bare StrAv-AuNP sample in the absence of biotin-LUVs (roughly 0.01% of the AuNPs detected in NTA) displays signals that are not easily distinguishable from complexes formed between StrAv-AuNPs and biotin-LUVs, even after optimized functionalization with StrAv (Figure 4). Yet, considering the multiparametric information obtained from the integrated real and integrated imaginary part of the optical signal, as well as size, the probability that a detected object does not originate from the presence of biotin-LUVs is estimated to be less than 3%, when the analysis is gated for integrated imaginary and real values below −1.3 and above 0.6, respectively (Figure 4). Achieving this at sub-pM concentrations of biotin-LUVs after approximately 30 min of incubation circumvents the need to adjust the AuNP concentration to match that of the entities to be detected, which inevitably results in reduced reaction times in the low concentration regime.

These results also hold potential for similar detection limits of optically faint biological nanoparticles, such as viruses, as previously reported,^19^ and extracellular vesicles, even in complex media. Achieving the latter will likely require employing high-molecular-weight PEG modifications as explored in this study, which have been demonstrated to more effectively suppress nonspecific biomolecular adsorption compared to self-assembled monolayers produced using shorter PEG.^43^ Extending off-axis holography to detect complex formations between AuNPs and biological nanoparticles in complex media will also require the attachment of alternative ligands, such as antibodies, aptamers, or enzymes, to PEG-modified AuNPs,^26^ typically utilizing a small fraction of PEG molecules modified with end groups suitable for chemical attachment to desired ligands.

In this case, as well as for the use of biotin functionalization of AuNPs in cancer treatment^28^ and ongoing efforts presently undertaken to apply similar PEG-based modifications to lipid nanoparticles for targeted gene delivery,^55^ the efficiency of the coupling chemistry is anticipated to be critically dependent on the relative lengths of active and nonactive PEG. It is also worth noting that efficient coupling chemistry can significantly reduce the need for high protein concentrations during the NP functionalization step, thereby enhancing the overall practicality and cost-effectiveness of the process when working with valuable biomolecular preparations.^19^

## Materials and Methods

### AuNP Fabrication

AuNPs with a 7 nm radius were fabricated by addition of 6 mL of 1 wt % sodium citrate (Sigma-Aldrich) to a mixture of 40 μL of 25% w/v HAuCl_4_ (Sigma-Aldrich) diluted in 100 mL of Milli-Q water and heated to 100 °C under vigorous stirring. The solution was left at the boiling point for 15 min and centrifuged in 2 mL aliquots at 2000*g* for 10 min, and the bottom 10% was discarded. AuNPs with an increased radius (25 nm) were subsequently produced by simultaneous addition of 1 mL of 30 mM hydroquinone (Sigma-Aldrich) and 220 μL of 1 wt% sodium citrate into a solution of 40 μL of 25% w/v HAuCl_4_ and 2.225 mL of 7 nm radius AuNPs diluted in 100 mL of Milli-Q water (approximately 6.5 × 10^10^/mL) under rapid stirring at room temperature. This mixture was left under moderate stirring for at least 1 h. Size distributions and concentrations were estimated using DLS (Zetasizer Ultra), NTA (NanoSight LM10), and UV–Vis spectroscopy (Jenway 6705) for both 7 and 25 nm AuNPs (Supporting Information, Figures S2 and S3).

After concentration through centrifugation (final concentration 2.1 × 10^12^/mL) in 1 mL aliquots at 1000*g* for 30 min and subsequent collection of the soft pellet, the 25 nm AuNPs were PEGylated by extensive mixing of 250 μL of AuNPs and a 250 μL solution of an approximately 0.3 nM solution of either 3 or 5 kDa thiol-PEG, with 0.06% SH-PEG-biotin, and incubated at 4 °C overnight. Excess PEG was removed through eight consecutive filtration steps using a 300 kDa centrifugal filter (Pall) at 400*g* for 17 min.

StrAv modification was performed by first mixing 400 μL biotin-PEG-AuNPs (milli-Q) with 10 μL bovine serum albumin (BSA, Sigma-Aldrich, 10 mg/mL) to a final biotin-PEG-AuNP concentration of ∼10^11^/mL. The biotin-PEG-AuNP suspension was then added to a StrAv (MP Biomedicals) suspension (PBS) with StrAv concentrations of either 17 nM, 170 nM, or 1.7 μM at 2 μL/min using a positive displacement pump (VICI M Series) with a tube with an inner diameter of 500 μm. This was followed by an identical filtration procedure to that used after PEGylation, and finally stored in a solution of PBS (pH 7.4) and 0.1 mg/mL BSA. Size distributions and concentrations were estimated using DLS, NTA, and UV–Vis spectroscopy for each step (Supporting Information, Figures S2 and S3).

### Biotin-LUV Fabrication

Lipid vesicles were fabricated of 99% 1-palmitoyl-2-oleoyl-glycero-3-phosphocholine (POPC) (Avanti Research) and 1% 1,2-distearoyl-*sn*-glycero-3-phosphoethanolamine-*N*-[biotinyl­(polyethylene glycol)-2000] (DSPE-PEG(2000) Biotin) (Avanti Research). A lipid film was formed in a glass vial from lipids dissolved in chloroform by evaporation under a nitrogen gas flow, followed by vacuum drying overnight. The film was rehydrated by the addition of PBS (pH 7.4) and rapid vortexing to a final lipid concentration of 2 mg/mL. Vesicles were produced by extrusion through a 400 nm pore size polycarbonate membrane filter (Whatman) 25 times.

### Nanoparticle Tracking Analysis

Size and concentration estimations were performed using a NanoSight LM10 NTA module (Malvern Instruments Ltd., 488 nm laser) at room temperature under a steady flow by a NanoSight syringe pump. Bare AuNPs (before concentration by centrifugation) and PEGylated AuNPs were diluted 1000× and 10,000× in Milli-Q water, corresponding to approximately 3 × 10^7^/mL and ∼10^8^/mL, respectively. For characterization, StrAv-AuNPs and biotin-LUVs were diluted 1000× and 5000× in PBS, corresponding to a concentration ∼10^8^ for both. Milli-Q water and PBS were both filtered using 0.02 μm syringe filters (Whatman Anotop 25) and degassed in vacuum.

For analytical measurements (Figure 1c), StrAv-AuNPs and biotin-LUVs were instead diluted to 4 × 10^8^/mL and 3 × 10^6^/mL, respectively, for separate measurements. For the measurement of the StrAV-AuNP/biotin-LUV mixture, the particles were mixed at 6.6 × 10^10^/mL and 5 × 10^8^/mL in a 30 μL sample and incubated at room temperature for 30 min before an additional 167× dilution.

### Off-Axis Holography

The twilight off-axis holographic microscope (Figure 5) was set up in accordance with Olsén et al.,^19^ with a mounted microfluidic 800 × 20 × 58.5 × 10^3^ μm (WxDxL) straight channel chip (microfluidic ChipShop) coated with 5 mg/mL bovine serum albumin. 6 μL of biotin-LUVs, corresponding to 5 × 10^8^/mL, was mixed with 24 μL of PBS (150 nM, pH 7.4). Approximately 0.6–1.3 μL of StrAv-AuNPs (depending on the sample), corresponding to 6.6 × 10^10^/mL, was mixed with PBS to a total volume of 30 μL. Moreover, biotin-LUVs and StrAV-AuNPs were mixed together in PBS, at a total volume of 30 μL and final concentrations of 5 × 10^8^/mL and 6.6 × 10^10^/mL, respectively, and incubated at room temperature for 30 min. Concentrations were determined by using NTA for biotin-LUVs and UV–Vis spectroscopy for AuNPs. All samples were measured under flow in three videos of 1500 frames at 41 frames/s. The flow rate was controlled by the addition of PBS to the outlet. Data analysis was performed in accordance with Olsén et al.^19^

**Figure 5:**
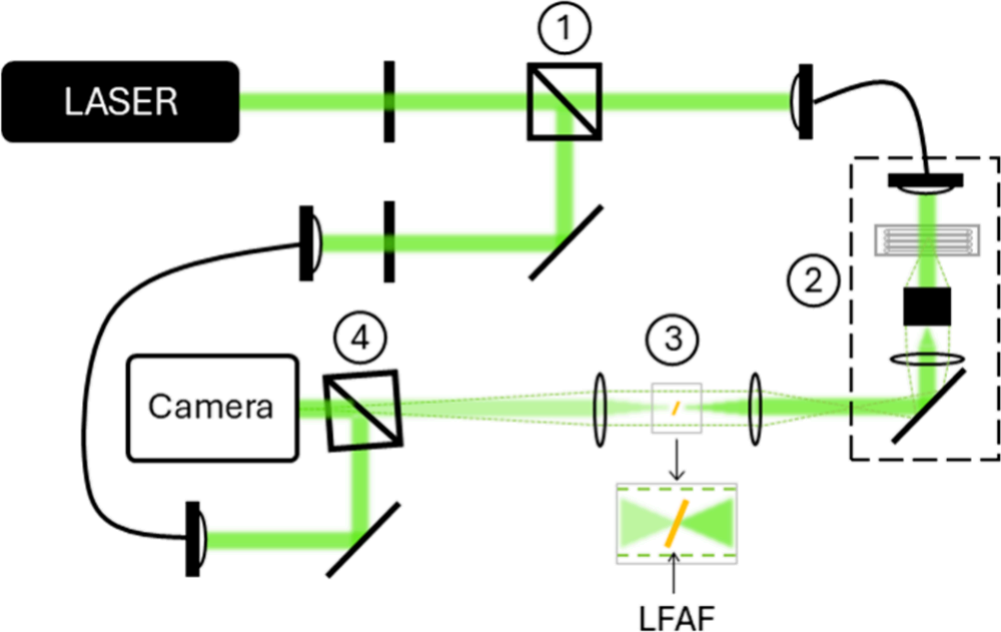
Schematic illustration of a twilight off-axis holographic microscope setup. Light from a DPSS laser (532 nm, Roithner Lasertechnik GmbH) is propagated through a beamsplitter (1) and the object beam continues through the sample and objective (2), followed by a low-frequency attenuation filter (LFAF) placed in the Fourier plane of a 4f system. The object and reference beams are recombined at the camera at an offset angle (4).

### Waveguide Scattering Microscopy

Waveguide chips (dimensions: length 8 mm, width 2 mm, height 35 μm) were cleaned by 5 min oxygen plasma treatment, sealed with a microfluidic channel, mounted in an Olympus BX61 microscope, then rinsed with 1% Hellmanex, Cobas, and Milli-Q water, and functionalized by 0.1 mg/mL PLL-*g*-PEG with 10% under a 10 μL/min flow for 10 min. After rinsing with 100 μL of degassed PBS, ∼10^9^/mL StrAv-AuNPs were sequentially injected at 10 μL/min for 1 min followed by PBS at 10 μL/min for 10 min and then by approximately 15 min of additional rinsing. Videos were recorded at 0.5 frames/s for 300 frames.

### Surface Plasmon Resonance

A gold-coated SPR chip was cleaned in accordance with the RCA-1 cleaning protocol (5:1:1 of H_2_O:NH_4_OH:H_2_O_2_) at 75 °C for 15 min and left in Milli-Q water for at least 1 h. The chip was stored in 99.7% EtOH, dried under nitrogen gas flow, and mounted in an SPR instrument (BioNavis Navi 220A). The chip was functionalized with 99% α-hydroxy-ω-mercapto PEG (Rapp Polymere) and 1% biotin-PEG-thiol (Nanocs) at 1 nM in Milli-Q water and flowed at 10 μL/min for 40 min. After rinsing, the Milli-Q water was replaced by vacuum-degassed PBS and streptavidin samples at 1.7 nM, 8.5 nM, 17 nM, 170 nM, and 1.7 μM were introduced sequentially at 20 μL/min for 5 min, with a PBS rinsing step between each sample.

### UV–Vis Spectroscopy

Gold nanoparticle suspensions were diluted in 200 μL samples such that the absorbance was approximately between 0.3 and 0.5, corresponding to 2-fold and 200-fold dilution in Milli-Q water for bare AuNPs (before concentration by centrifugation) and concentrated bare AuNPs as well as PEGylated AuNPs, respectively, and a 10-fold dilution in PBS for StrAv-AuNPs. For 25 nm AuNPs, 0.3–0.5 absorbance corresponds to 8 × 10^9^ to 2 × 10^10^/mL. 7 nm AuNPs were diluted 5×, corresponding to 2.4 × 10^11^/mL. Measurements were performed using a quartz microcuvette (Hellma) and a Jenway 6705 ultraviolet–visible spectrophotometer.

### Dynamic Light Scattering

Size distributions were estimated using dynamic light scattering performed on a Zetasizer Ultra (Malvern Panalytical) at room temperature in backscatter mode using disposable PMMA cuvettes (Brand). Seed AuNPs, bare AuNPs (before concentration by centrifugation), and PEGylated AuNPs were diluted 100×, 50×, and 4000× in Milli-Q water, while StrAv-AuNPs were diluted 500× in PBS, to a total volume of 1 mL. These dilutions correspond to ∼10^8^/mL. For each measurement, default measurement settings were used.

## Supplementary Material















## Notes

Supplementary figures and sections cited in the main text: AuNP aggregation during surface modification measured using twilight off-axis holography (Figure S1), characterization of AuNPs throughout the fabrication steps using DLS and UV–Vis spectroscopy (Figures S2 and S3), evaluation of plasmonic shifts of of complex formation between StrAv-AuNP and biotin LUVs using UV-Vis spectroscopy article characterization (Section S2), derivation of eqs 1 and 2 (Figure S4) (PDF)
(Video S1) StrAv-(5k/5k)­PEG-AuNPs (Prep. Low) on the biotinylated PLL-*g*-PEG surface measured using WGSM (MP4)
(Video S2) StrAv-(5k/5k)­PEG-AuNPs (Prep. Med.) on the biotinylated PLL-*g*-PEG surface measured using WGSM (MP4)
(Video S3) StrAv-(5k/5k)­PEG-AuNPs (Prep. High) on the biotinylated PLL-*g*-PEG surface measured using WGSM (MP4)
(Video S4) StrAv-(5k/3k)­PEG-AuNPs (Prep. Low) on the biotinylated PLL-*g*-PEG surface measured using WGSM (MP4)
(Video S5) StrAv-(5k/3k)­PEG-AuNPs (Prep. Med.) on the biotinylated PLL-*g*-PEG surface measured using WGSM (MP4)
(Video S6) StrAv-(5k/3k)­PEG-AuNPs (Prep. High) on the biotinylated PLL-*g*-PEG surface measured using WGSM (MP4)
