# Supplementary material for: Quantitative Detection of Biological Nanoparticles Using Twilight Off-Axis Holographic Microscopy: Insights on Complex Formation between PEGylated Gold Nanoparticles and Lipid Vesicles
Source: J Phys Chem B. 2025 Sep 9;129(37):9506–16. doi: 10.1021/acs.jpcb.5c04228 (PMC12451665; doi:10.1021/acs.jpcb.5c04228)
Supplement: Supplementary file 1 [file jp5c04228_si_001.pdf]

Supplementary Information

Quantitative Detection of Biological Nanoparticles Using Twilight Off-Axis Holographic Microscopy: Insights on complex formation between PEGylated Gold Nanoparticles and Lipid Vesicles

Julia Andersson[1], Anders Lundgren[2], Erik Olsén[3], Petteri Parkkila[1] Daniel Midtvedt[4], Björn Agnarsson[1], and Fredrik Höök[1#]

1) Chalmers University of Technology, Department of Physics, Division of Nano and Biophysics, Fysikgränd 3, 41296 Göteborg, Sweden

2) Department of Chemistry & Molecular Biology, University of Gothenburg, Medicinaregatan 7b, 41390 Göteborg

3) Michael Smith Laboratories, University of British Columbia, 2185 East Mall, Vancouver, V6T 1Z4, BC, Canada

4) Department of Physics, University of Gothenburg, Origovägen 6b, 41296 Göteborg

S1. Gold Nanoparticle Aggregation after PEGylation and Streptavidin modification

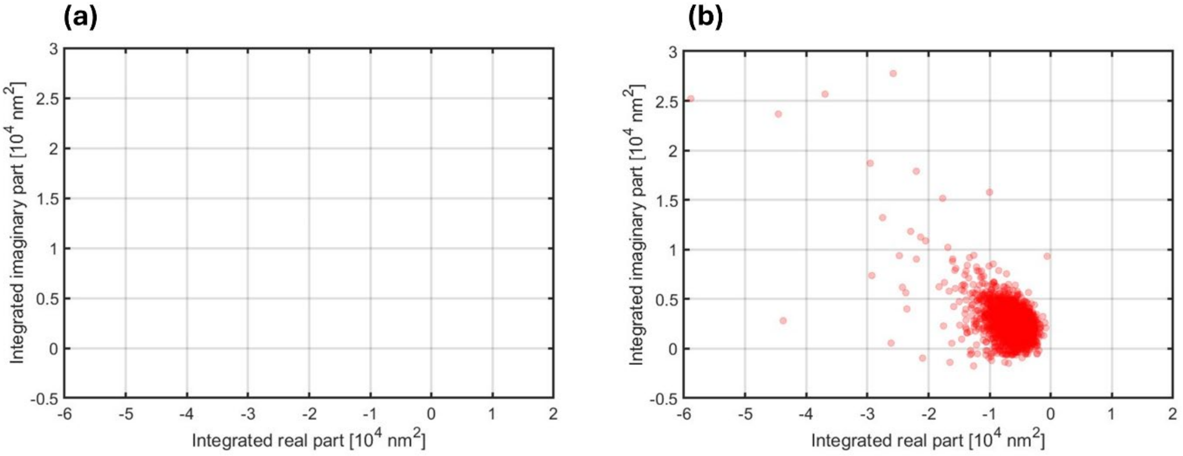

FIgure S1: Evaluation of aggregation of (a) biotin-PEG(5k)-AuNPs in milli-q water and (b) StrAv-AuNPs functionalized with approximately 2 StrAV moieties/AuNPs in PBS. It is apparent that AuNP aggregation occurs during StrAv modification.

S2. Gold Nanoparticle Characterization

AuNPs were fabricated and functionalized in four step: seed synthesis, seed-mediated growth, PEGylation and StrAv-modification. After each step, the size distribution and concentration was estimated using nanoparticle tracking analysis (NTA), dynamic light scattering (DLS) and ultraviolet-visible spectorscopy (UV-Vis). UV-Vis and DLS results are presented in Figs. S2 and S3, respectively, while the NTA results are shown in Figs. 1 and 2. Note that the AuNP seeds were not measured using NTA, as they fall below the detection limit.

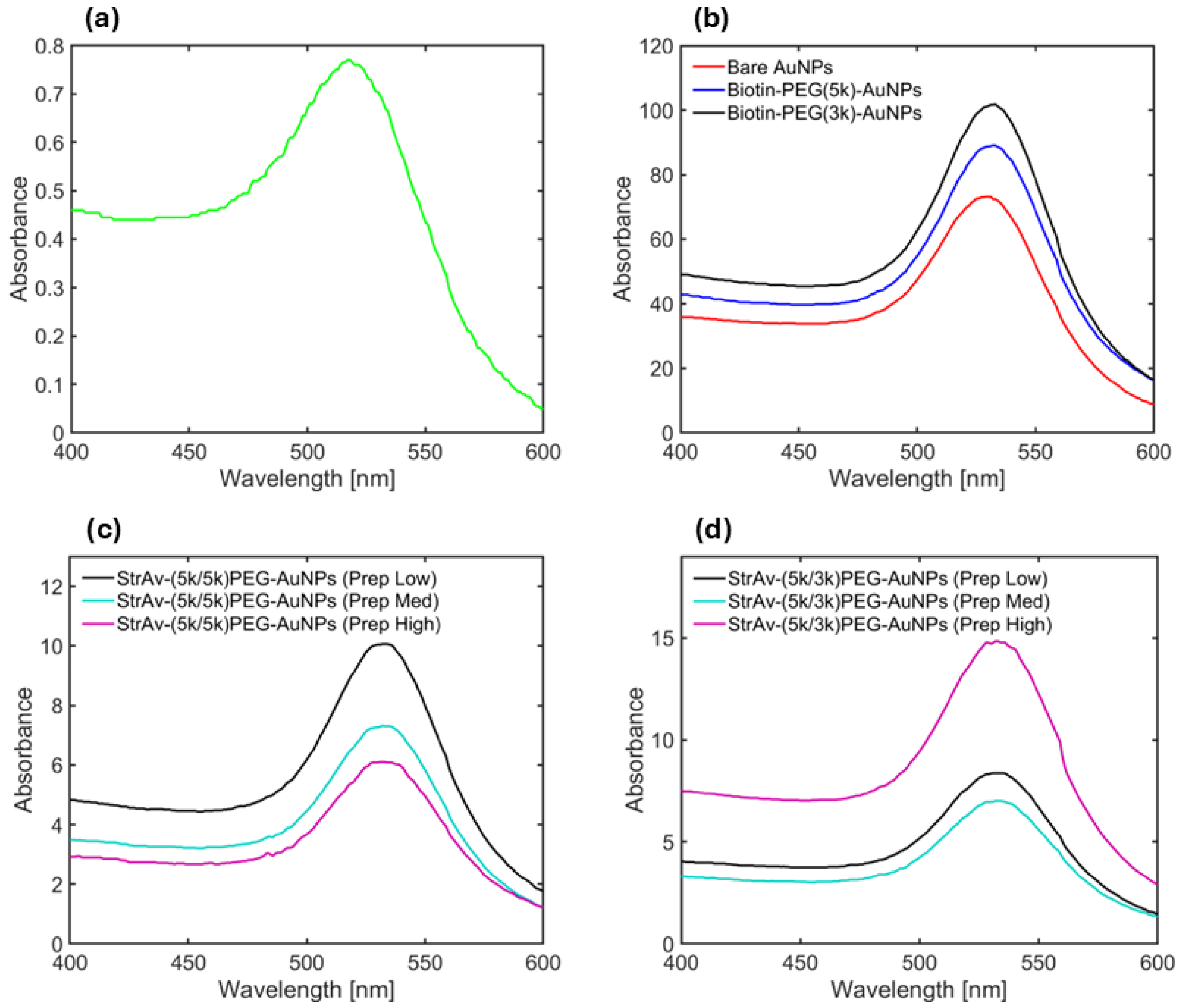

Figure S2. The absorbance spectra of **(a)** AuNPs seeds, **(b)** fully grown, bare AuNPs as well as PEGylated AuNPs, and StrAv-AuNPs functionalized with either **(c)** 5 kDa SH-PEG or **(d)** 3 kDa SH-PEG and 0.06% 5 kDa SH-PEG-biotin were evaluated using UV-Vis spectroscopy. The spectra have been adjusted to account for dilution. By these, both concentration and particle size can be estimated.

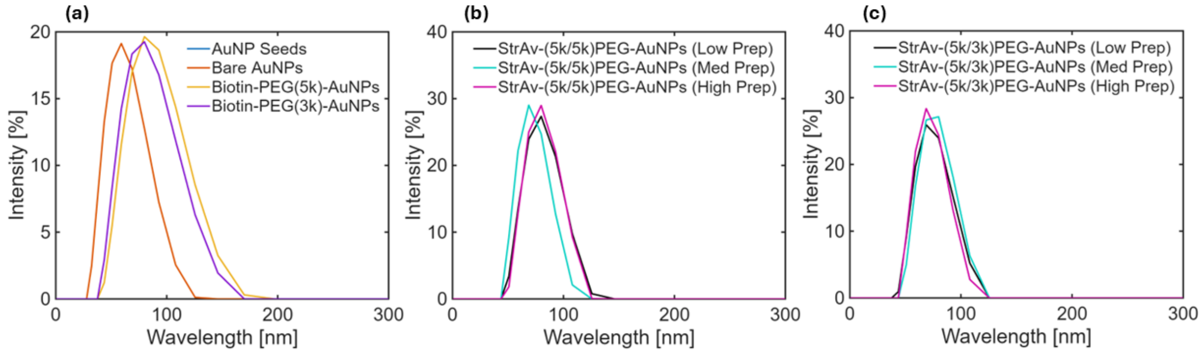

Figure S3. The size distribution of **(a)** AuNP seeds, fully grown bare AuNPs and PEGylated AuNPs, as well as StrAv-AuNPs functionalized with either **(b)** 5 kDa SH-PEG or **(c)** 3 kDa SH-PEG and 0.06% 5 kDa SH-PEG-biotin were evaluated using DLS.

S3. Evaluation of Plasmonic Coupling of Gold Nanoparticles in Complex with Biotinylated Lipid Vesicles

To evaluate whether StrAv-AuNPs display any plasmonic coupling once bound to biotin-LUVs, as would be indicated by plasmonic broadening or peak shifting, StrAv-AuNPs were measured using UV-Vis spectroscopy both individually and in mixture with biotin-LUVs at varying StrAv-AuNP to biotin-LUV ratios (Fig. S4). Here, a StrAv-AuNP to biotin-LUV ratio of 1564:1 corresponds to that used for the off-axis holography measurements, while the additional graphs denote a further dilution of the LUVs by a factor of 10 and 50, respectively. All samples were incubated at room temperature for 30 min, at concentrations corresponding to the off-axis holography measurements outlined in the main text, before being further diluted to comply with the operating range of the UV-Vis instrument. As evidenced by the lack the negligible difference between the plasmonic peaks (Fig. S4), neither plasmonic coupling between the StrAv-AuNPs, nor colorimetric shifts due to a change in refractive index near the AuNP surface were observed.

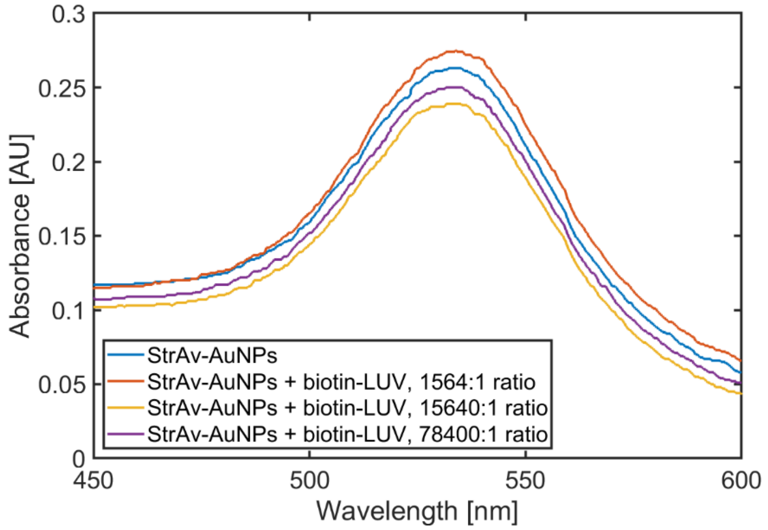

Figure S4. Measurement of StrAv-AuNPs (blue) as well as mixtures of StrAv-AuNP with biotin-LUVs at a ratio of 1564:1 (red), 15640:1 (yellow) and 78400:1 (purple). All samples were mixed at a StrAv-AuNP concentration of 6.6×10^{10}/mL, then diluted after 30 min incubation to comply with the operating range of the UV-Vis spectrophotometer.
